# Meiosis and beyond – understanding the mechanistic and evolutionary processes shaping the germline genome

**DOI:** 10.1111/brv.12680

**Published:** 2021-01-01

**Authors:** Roberta Bergero, Peter Ellis, Wilfried Haerty, Lee Larcombe, Iain Macaulay, Tarang Mehta, Mette Mogensen, David Murray, Will Nash, Matthew J. Neale, Rebecca O'Connor, Christian Ottolini, Ned Peel, Luke Ramsey, Ben Skinner, Alexander Suh, Michael Summers, Yu Sun, Alison Tidy, Raheleh Rahbari, Claudia Rathje, Simone Immler

**Affiliations:** ^1^ Institute of Evolutionary Biology University of Edinburgh Edinburgh EH9 3JT U.K.; ^2^ School of Biosciences University of Kent Canterbury CT2 7NJ U.K.; ^3^ Earlham Institute Norwich Research Park Norwich NR4 7UZ U.K.; ^4^ Applied Exomics Ltd Stevenage Bioscience Catalyst Stevenage SG1 2FX U.K.; ^5^ School of Biological Sciences University of East Anglia Norwich Research Park Norwich NR4 7TJ U.K.; ^6^ Genome Damage and Stability Centre, School of Life Sciences University of Sussex Brighton BN1 9RH U.K.; ^7^ The Evewell 61 Harley Street London W1G 8QU U.K.; ^8^ The James Hutton Institute Invergowrie Dundee DD2 5DA U.K.; ^9^ School of Life Sciences University of Essex Colchester CO4 3SQ U.K.; ^10^ Department of Organismal Biology Uppsala University Norbyvägen 18D Uppsala 752 36 Sweden; ^11^ The Bridge Centre 1 St Thomas Street, London Bridge London SE1 9RY U.K.; ^12^ Norwich Medical School University of East Anglia Norwich Research Park, Colney Ln Norwich NR4 7UG U.K.; ^13^ School of Biosciences University of Nottingham, Plant Science, Sutton Bonington Campus Sutton Bonington LE12 5RD U.K.; ^14^ Wellcome Sanger Institute Hinxton Cambridge CB10 1SA U.K.

**Keywords:** recombination, mutation rate, DNA repair, double‐strand breaks, mutation hotspots, recombination hotspots, selection

## Abstract

The separation of germ cell populations from the soma is part of the evolutionary transition to multicellularity. Only genetic information present in the germ cells will be inherited by future generations, and any molecular processes affecting the germline genome are therefore likely to be passed on. Despite its prevalence across taxonomic kingdoms, we are only starting to understand details of the underlying micro‐evolutionary processes occurring at the germline genome level. These include segregation, recombination, mutation and selection and can occur at any stage during germline differentiation and mitotic germline proliferation to meiosis and post‐meiotic gamete maturation. Selection acting on germ cells at any stage from the diploid germ cell to the haploid gametes may cause significant deviations from Mendelian inheritance and may be more widespread than previously assumed. The mechanisms that affect and potentially alter the genomic sequence and allele frequencies in the germline are pivotal to our understanding of heritability. With the rise of new sequencing technologies, we are now able to address some of these unanswered questions. In this review, we comment on the most recent developments in this field and identify current gaps in our knowledge.

## I. INTRODUCTION

**Fig 1:**
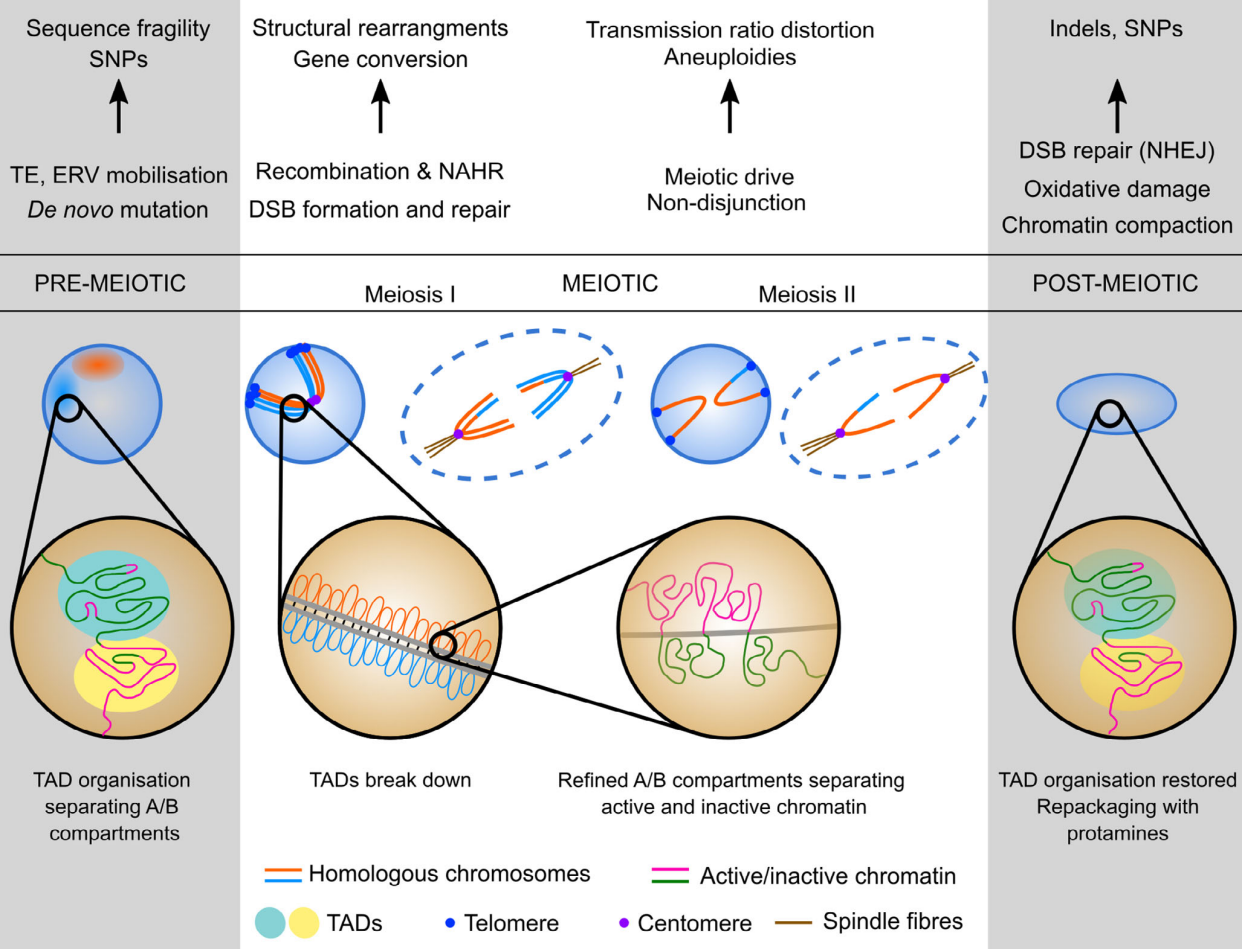
Illustration of genomic reconfiguration during spermatogenesis, and the processes that can lead to mutational outcomes. Prior to meiosis, chromatin is organised into topologically associated domains (TADs). These TADs are lost during prophase I, wherein the chromatin is separated more distinctly into active and inactive compartments despite its aggregation into a linear array of loops bound along a proteinaceous chromosome axis. Mobilisation of transposable elements (TEs) and endogenous retroviruses (ERVs), along with programmed recombination depending on the topoisomerase‐related enzyme, meiotic recombination protein SPO11 and nuclear division, provide opportunities for structural genomic changes *via* non‐allelic homologous recombination (NAHR) or non‐dysjunction, while the repair of DNA double‐strand breaks (DSBs) at the post‐meiotic stage also allows smaller indels and single nucleotide polymorphisms (SNPs) to arise *via* the non‐homologous end joining (NHEJ) pathway. Data from Alavattam *et al*. (2019), Patel *et al*. (2019) and Wang *et al*. (2019).

The evolution of a germline as a consequence of multicellularity has several far‐reaching consequences for biology in general and for genetics more specifically. One key aspect is that only genetic information present in the germ cells can be passed on to future generations and is exposed to evolutionary processes at the macro‐evolutionary scale. Many of the concepts that we currently study in population genetics and genomics apply to germ cell populations. Any events affecting the genetic composition and variation in germ cells, including (*i*) in the pre‐meiotic germ cells leading up to meiosis, (*ii*) during meiotic division and recombination events, and (*iii*) in the haploid gametophytes and gametes after meiosis, have the potential to affect and alter offspring genotypes. Any irregularities in these complex processes may have major consequences not only for the organism itself, in terms of reducing its reproductive success, but also for subsequent generations. These events include germline mutations (e.g. Chen *et al*., 2017) and germline selection (Hastings, 1991) occurring during mitotic germ cell growth and proliferation, genome reorganisation, the separation of the chromosomes, recombination events occurring during meiosis (e.g. Schwarzacher, 2003; Ohkura, 2015), as well as mutations and selection affecting genetic variation at post‐meiotic stages (Immler & Otto, 2018; Fig. 1). Consequently, these events have potentially major implications for evolutionary processes and also have direct clinical and other applications. Despite substantial variation in reproductive modes across taxa, the processes before, during and after meiosis are remarkably conserved at the genomic level (Ohkura, 2015). While the genetic consequences of meiotic recombination have been considered in several excellent reviews (e.g. Lenormand *et al*., 2016; Stapley *et al*., 2017), herein we provide an overview of current knowledge of the genomic mechanisms involved before, during and after meiosis that may contribute to genetic variation among germ cells and thereby to populations. We specifically focus on the micro‐evolutionary processes known to affect inheritance and genetic variation, namely mutation, recombination and selection.

Germline mutation, both pre‐ and post‐meiotic, is the foundation of all inherited genetic variation, providing the substrate upon which both neutral and selective evolutionary processes act. Mutagenic processes are non‐random and exhibit substantial heterogeneity in the rate and type of mutations that occur, dependent on the local sequence context. This biased mutation spectrum has the power to shape not only neutral but also adaptive evolutionary trajectories (Stoltzfus & McCandlish, 2017). An accurate characterisation of germline mutation processes thus informs all aspects of evolutionary genomic analysis and is essential both for the accuracy of molecular phylogenetic reconstructions and for understanding the origins of inherited disease. Mutational biases are rooted in the detailed biochemistry of DNA synthesis, damage and repair. To date, much of our knowledge derives from the analysis of somatic mutations arising during mitosis in unicellular organisms (Buss, 1987; Hastings, 1989; Otto & Hastings, 1998; Flatt *et al*., 2008), in cell lines (Haldane, 1947; Li, Yi & Makova, 2002; Ermolaeva *et al*., 2013), in cancer cells during tumorigenesis (Haldane, 1947; Gupta *et al*., 1989; Alexandrov *et al*., 2020) or from mutation‐accumulation experiments (reviewed in Halligan & Keightley, 2009). Collectively, these studies have yielded a wealth of information on the ‘mutational signatures’ associated with different DNA‐damaging agents, different DNA damage‐repair pathways, and the effect of genetic lesions affecting each pathway. Mutational spectra during gametogenesis have been studied less intensively than those of other cell types due to the difficulty of genotyping multiple parent/offspring trios to identify *de novo* mutations, however, some of the same signatures have been observed in these studies and/or inferred from the population distribution of mutations in autozygous genome segments (UK10K Consortium *et al*., 2016; Narasimhan *et al*., 2017). Here, we focus on the unique environment of the germline, and how this influences the mutational signatures observed during gametogenesis.

Recombination is a key meiotic process contributing to genetic variation and trait inheritance thereby playing a key role in trait evolution. Understanding the recombination landscape in a genome allows predictions about the heritability of traits and of the co‐evolutionary dynamics between traits. Linkage maps have been published for many species, with representative taxa ranging from yeast to plants and humans (e.g. Meinke, Sweeney & Muralla, 2009; Wang *et al*., 2011, 2012; Acquaah, 2012; Baryshnikova *et al*., 2013). However, most of these maps were built from sequencing information obtained from parents and offspring and/or population‐level data (International HapMap Consortium, 2005; Kong *et al*., 2010) and depend strongly on the number of individuals sequenced. More recently, single‐cell sequencing in sperm and eggs has provided personalised recombination maps for both sexes (Wang *et al*., 2012; Hou *et al*., 2013). Increasingly accurate sequencing with the help of long‐read sequencing technologies will contribute further to high‐resolution individual linkage maps.

Finally, selection in the germline may occur at any point from the mitotically dividing germ cells, to mechanisms influencing segregation and recombination events during meiosis (meiotic drive; Burt & Trivers, 2006) to post‐meiotic haploid stages (Joseph & Kirkpatrick, 2004; Immler & Otto, 2018; Immler, 2019). Selection events at these stages are likely to have profound effects on the heritability of specific alleles and alter allele frequencies at a much faster rate than any selection occurring at the population level. Possible mechanisms of pre‐meiotic germline selection may be *de novo* mutations occurring in germ cells that spread readily through the germline by increasing the proliferation rate of the cells that carry them. The mutation allele thus is found in a higher proportion of the resulting gametes than expected under a scenario without positive selection and hence has a higher heritability. Similarly, meiotic drive mechanisms can affect the relative frequency of a mutation in the resulting gametes either by successfully driving it towards the oocyte in female meiosis, e.g. *Kindr* in maize (*Zea mays*) (Dawe *et al*., 2018) and satellite drive (Akera, Trimm & Lampson, 2019) in the house mouse (*Mus musculus domesticus*); or by outcompeting wild‐type alleles during male meiosis, e.g. Paris sex ratio drive (Helleu *et al*., 2016) or during male post‐meiosis, e.g. *t*‐haplotype (Lyon, 2003) in the house mouse, and *Segregation Distorter* (*SD*) gene complex (Larracuente & Presgraves, 2012) and Winters (Tao *et al*., 2007) in *Drosophila* fruitflies. Finally, selection among haploid gametes may strongly affect the genetic composition of the next generation (e.g. Alavioon *et al*., 2017; Borowsky, Luk & Kim, 2018; Immler & Otto, 2018; Rathje *et al*., 2019). Most such ideas of selection occurring between two generations are still poorly explored and require additional attention in order to improve our understanding of inheritance and evolution.

Herein, we provide an overview of the latest insights into the processes shaping the germline genome before, during and after meiosis. We identify gaps in our knowledge, linking our understanding of the mechanistic processes affecting the germline genome with their evolutionary significance and considering how recently developed and future technologies may help in elucidating the underlying mechanisms contributing to these processes. The different sections vary markedly in depth and detail, largely due to the current level of knowledge we have about the various stages.

Mechanistic processes involved in meiotic stages have been studied in great detail and we direct the reader to several excellent reviews (Mercier *et al*., 2016; Lenormand *et al*., 2016; Stapley *et al*., 2017). However, for other aspects, such as the pre‐ and post‐meiotic stages, we are only at the very beginning of understanding their evolutionary importance.

## II. PRE‐MEIOTIC PHASE

Mitotic divisions during pre‐meiotic proliferation are a key stage for possible change and damage affecting the germline genome and thus genomes in the following generation. Molecular mechanisms have evolved specifically to protect and maintain the germline genome at these stages (Bloom *et al*., 2019). However, evidence is increasing that the genome experiences substantial changes during mitotic divisions, which contribute to the genomic information passed on to subsequent generations. These effects include the occurrence of *de novo* mutations and possible selection among mitotic germ cells resulting in deviations from Mendelian inheritance.

### (1) Pre‐meiotic mutations

In mitotically dividing early germ cells, mutational processes are similar to those occurring in somatic cell types, and largely reflect ubiquitous error sources such as base misincorporation during DNA synthesis and single base changes caused by deamination. However, the segregation of the germline from the soma in metazoans means that even non‐specific processes occurring in the germline can lead to mutational signatures when observed over evolutionary timeframes. These biases are well established in animals and some fungi but are less characterised in plants owing to their later segregation of germ cells from the somatic lineages (Schmid‐Siegert, Sarkar & Reymond, 2017). Here, we discuss three such cases: (*i*) DNA synthesis errors, (*ii*) C‐to‐T transitions, and (*iii*) transcription‐associated changes.

Errors occurring during DNA replication result in mutations, i.e. changes in DNA sequences. Although most germline mutations are at least mildly deleterious and can be the primary cause of heritable diseases, they are also the primary source of genetic variation and consequently of evolutionary adaptation. The two main types of mutations are nucleotide substitutions and small insertions/deletions (indels). Nucleotide substitutions occur roughly 10 times more frequently than indels during germline cell replication. Genome‐wide studies place the germline *de novo* mutation rate per nucleotide per generation at 1.2 × 10^−8^ in humans, and 5.7 × 10^−9^ in mice (Milholland *et al*., 2017). DNA synthesis errors accumulate according to the number of cell divisions per generation. Consequently, one of the most prominent germline mutational signatures – an increased proportion of paternally inherited *versus* maternally inherited mutations – is in part explicable as a consequence of the greater number of cell divisions involved in male reproduction (Haldane, 1947; Wilson Sayres & Makova, 2011; Jónsson *et al*., 2017; Wu *et al*., 2020; but see also Gao *et al*., 2018).

In order to suppress retroelement proliferation (see Section II.2), male and female germ cells show gains in cytosine methylation (Walsh, Chaillet & Bestor, 1998). However, methylated cytosine can spontaneously mutate to thymine through deamination (Sved & Bird, 1990). Because methylated cytosine exists primarily in the dinucleotide CG (or CpG) throughout the animal kingdom, this long‐term C‐to‐T transition leads to depletion of CpG in genomic regions subject to germline cytosine methylation. Conversely (in mammals), demethylation associated with transcriptional activity leads to a characteristic relative enrichment of the CpG dinucleotide in ‘CpG islands’ at the promoters of housekeeping genes and genes expressed specifically in the germline, but not at other tissue‐specific gene promoters (Ponger, Duret & Mouchiroud, 2001; Sarda *et al*., 2012).

Similarly, inefficient repair of DNA damage can lead to biased mutation patterns based on germline transcriptional activity (Xia *et al*., 2018; Seplyarskiy *et al*., 2019). This mechanism of DNA repair relies on the RNA polymerase stalling at and recognising a DNA lesion on the transcribed DNA strand, and typically produces biased mutation patterns because genes not expressed in the germline cannot benefit from this transcription‐coupled repair (TCR). Genes not expressed in the testes typically display higher mutation rates than actively transcribed genes, show higher sequence diversity in populations and diverge faster over evolutionary timescales. We predict that biased mutation patterns could also occur for other strand‐specific processes such as APOBEC (apolipoprotein B mRNA editing enzyme, catalytic polypeptide‐like) deamination (Haradhvala *et al*., 2016).

Despite strong conservation in the DNA replication machinery, considerable variation in mutation rates and patterns both within and among species has been reported recently (Chintalapi & Moorjani, 2020). Mutation rates vary by orders of magnitude across vertebrates, and within primates there is an almost twofold difference in yearly substitution rates between apes and monkeys. Previous work has proposed that differences in germline mutation processes among species depend on life‐history traits (Amster & Sella, 2016; Gao *et al*., 2016), but a recent study that compared germline mutations between humans and mice suggested that variation in mutation rates has a cellular and molecular basis (Lindsay *et al*., 2019). Another view is that mutation rates could be affected by sexual selection, as a male bias in mutation rates has been observed across a variety of animal species (Ellegren, 2007; Wilson Sayres & Makova, 2011), and a recent study on male seed beetles *Callosobruchus maculatus* seems to support this (Baur & Berger, 2020). Pedigree‐based studies with different regimes of natural and sexual selection, facilitated by advances in genome sequencing, should be carried out to establish a truly comprehensive understanding of the variation in germline mutation processes across species.

A male bias in mutation rates also occurs in humans: most germline mutations are paternal in origin; the number of mutations increases significantly with paternal age but only weakly with maternal age. Interestingly, it has also been suggested that the mutation rate has changed over the course of human evolution. This is based on the observation that the yearly mutation rate estimated from human pedigree studies is almost half the rate inferred by looking at sequence divergence between human and chimpanzee (*Pan troglodytes*) genomes, suggesting a potential slowdown in mutation rate in humans (Scally & Durbin, 2012; Besenbacher *et al*., 2019). By contrast, in olive baboons (*Papio anubis*) the mutation rate appears to be lower than in humans (Wu *et al*., 2020).

### (2) Transposon/retrovirus mobilisation

A further germline‐specific source of mutational novelty is mobilisation of transposable elements (TEs) and endogenous retroviruses (ERVs), selfish genetic elements that require germline‐specific (retro)transposition in order to increase their fitness. Although TEs, ERVs, and remnants thereof make up significant proportions of genomes, for example nearly half of the human genome (Cordaux & Batzer, 2009) and up to 80% in some grass species (Barakat, Carels & Bernardi, 1997), only a few are able to mobilise (Brouha *et al*., 2003; Cordaux, Hedges & Batzer, 2004). Most knowledge on TE and ERV mobilisation derives from cell lines, cancer cells, and somatic development (Boeke *et al*., 1985; Huang, Burns & Boeke, 2012; Nätt & Thorsell, 2016) and has been complemented only in some model systems by direct studies of germline‐specific mobilisation [e.g. mouse and fruitfly (Timakov *et al*., 2002; Newkirk *et al*., 2017; Richardson & Faulkner, 2018)]. Three major mechanisms of (retro)transposition have been identified: (*i*) cut‐and‐paste transposition of DNA transposons, (*ii*) target‐primed reverse transcription (TPRT) of long and short interspersed elements (LINEs and SINEs), and (*iii*) replicative retrotransposition of ERVs and long terminal repeat (LTR) retrotransposons (Levin & Moran, 2011).

Cut‐and‐paste transposition involves precise excision of a DNA transposon by its transposase and reinsertion at a new genomic locus. One type of cut‐and‐paste transposable elements known as P elements seem preferentially to insert in promoter regions of the male germline genome of *Drosophila melanogaster*, but not in germline genomes of wild females because of an inhibitor of transposase activity specifically expressed in females (Timakov *et al*., 2002). Another important mechanism to counteract cut‐and‐paste transposition in the germline has been identified in *Caenorhabditis elegans*. In this model organism, Tc1/*mariner* transposons can jump in the soma, but not in the germline genome because of a mechanism of transposon silencing, which specifically uses a mechanism of RNA interference (Vastenhouw & Plasterk, 2004). Interesting insights on the germline *versus* soma dynamics of mobile elements come from a study on *Tetrahymena thermophila*, a binucleate ciliate that possesses two types of nuclei, a germline micronucleus and a somatic macronucleus (Feng *et al*., 2017). During the sexual phase of this ciliate, a transition from micro‐ to macronucleus occurs *via* a complex programmed genome rearrangement, with chromosome fragmentation and elimination of internal chromosome regions. This is regarded as a genome defence mechanism, since the eliminated sequences are densely populated with transposon‐related sequences, which are excised by a *piggyBac* transposase with germline‐limited activity (Feng *et al*., 2017).

Non‐LTR retrotransposons duplicate through RNA intermediates which are imported into the nucleus, reverse transcribed into complementary DNA (cDNA) and inserted into a new genomic location by endonuclease nicking of the new genomic target site (Luan *et al*., 1993). The retrotransposon LINE‐1, *Alu* SINEs and SVAs (SINE‐VNTR‐Alu retrotransposons) are the only TEs active in modern human genomes, and account for one‐third of the human genome (Cordaux & Batzer, 2009). Their ability to move within the human, and more generally primate, genome has shaped genome and transcriptome evolution in this group over the past 80 million years through several mechanisms, including genomic rearrangement, ectopic recombination, genetic innovation *via* transduction‐mediated gene and exon formation, changes in expression of nearby genes, RNA editing and epigenetic regulation.

LINE‐1 mobilisation in the mouse germline *via* RNA intermediates has been studied using high‐throughput sequencing and reporter transgene approaches (Newkirk *et al*., 2017; Richardson & Faulkner, 2018). These studies have found that L1 elements can be silenced by a PIWI (P‐element induced Wimpy)‐interacting RNA, that L1 retrotransposition can occur in early primordial germ cells, and that most *de novo* L1 insertions, detected across multiple generations of mice, are heritable, with a frequency of at least one heritable retrotransposition event per 10 births (Richardson & Faulkner, 2018).

Replicative retrotransposition of ERVs and LTRs involves reverse transcription of RNA inside virus‐like particles in the cytoplasm, nuclear reimport of integrase‐bound cDNA, followed by insertion into a new genomic target site (Levin & Moran, 2011). Most human LTR elements are inactive endogenous retroviruses (HERVs), inserted into the human genome around 25 million years ago. However, the most recently acquired human ERV, HERVK, still retains intact open reading frames and can be reactivated in early embryonic cells (Grow *et al*., 2015). LTRs with active transposition in the germline have been found in *D. melanogaster* (Huang *et al*., 2012), and in the male germline of regenerated plants (Fukai *et al*., 2010).

The global ‘resetting’ of epigenetic marks during germline development (Hajkova, 2011) commonly triggers at least the transcription, if not (retro)transposition, of TEs and ERV copies capable of mobilisation. However, the timeframe of their mobilisation is not only limited to germline development but can also occur during early meiosis (Timakov *et al*., 2002; Newkirk *et al*., 2017; Hunter, 2017; Richardson & Faulkner, 2018) and may even result in the repair of meiotic double‐strand breaks (DSBs) with retrotransposon cDNA (Ono *et al*., 2015). In addition to silencing *via* epigenetic marks such as CpG methylation and histone modification, there is an ever‐growing list of silencing mechanisms acting at various levels of the aforementioned (retro)transposition mechanisms (Goodier, 2016; Kapusta & Suh, 2017). For example, the PIWI‐interacting (piRNA) pathway relies on sequence‐specific interference with RNAs from TEs and ERVs (Ghildiyal & Zamore, 2009), and APOBEC deamination hypermutates DNA from ERVs and LTR retrotransposons (Knisbacher & Levanon, 2016). The high diversity of TEs and ERVs on the one side and silencing mechanisms on the other is best explained by continuous intragenomic arms races between them (Kapusta & Suh, 2017). As a result, the occasional successful TE or ERV proliferation can act as a substrate for nonallelic homologous recombination (NAHR; see Section III.6) between and within such repetitive sequences (Devos, 2002; Sen *et al*., 2006). They can even mediate large chromosomal rearrangements as suggested by the enrichment of evolutionary breakpoint regions for TEs and ERVs (Farré *et al*., 2016). Finally, as TE and ERV mobilisation depends on DSB formation by their own enzymatic machinery (i.e. transposase, endonuclease, or integrase), this leads to an overall increase in DSBs across the genome, many of which are not associated with actual (retro)transposition events but still contribute to genomic instability (Cordaux & Batzer, 2009).

Transposable elements have evolved to be active in the germline, but silent in the soma (Haig, 2016), their location in the genome typically is not random but under strict control by selection and their frequency is subject to genetic drift. Despite the fact that TEs are primarily regarded as selfish elements with deleterious effects, their mobilisation within genomes can allow the rewiring of gene regulatory networks and the supply of raw DNA material for the evolution of protein coding‐genes and non‐coding RNAs (Haig, 2016; Bourque *et al*., 2018). After such ‘domestication’, the recycling of TE genes has taken place to generate disparate new functions, which range from a role in placental development to involvement in somatic recombination events typical of the vertebrate immune system (Bourque *et al*., 2018). The rise of genomics has helped unravel the beneficial role of TEs, and will continue to bring important insights into their crucial role in genome evolution and in understanding the processes of genome diversification during population divergence and speciation.

### (3) Germline selection

The germline is founded by a single cell which continues to grow through mitotic divisions until sexual maturity of the organism, particularly in males. The mitotic proliferation of the germline generates a pool of genetically identical cells. However, mutations, mitotic cross‐overs and mitotic gene conversions can generate genetic diversity in this otherwise clonal line of diploid cells. Genetic variation among germ cells may result in selection for specific cell lineages. If such germ cell mutations have an effect on the efficiency of mitotic proliferation, their allelic frequencies in mature cells may deviate from expected frequencies under a neutral scenario (Crow, 2012). Such germline selection may either be beneficial if pre‐meiotic variation in cell line survival results in the effective removal of deleterious mutations, or may be disadvantageous if deleterious selfish genetic elements manage to promote the growth of the host germ cells in competition with others (Otto & Hastings, 1998).

In addition, genetic germline mosaicism may be associated with reduced fertility: a study investigating the genetic composition in the ejaculates of 100 men showed a low level of mosaicism in sex chromosomes in one third of men with fertility problems (Schiff *et al*., 2010). Mosaicism can also increase with paternal age. Achondroplasia is a common human disease associated with paternal age, caused in 95% of cases by *de novo* mutations occurring in sperm. Recent insights suggest that this increased frequency of mutations in older fathers is based on selection on spermatogonial stem cells, which change from the typical asymmetrical cell division to a symmetrical division, resulting in a more rapid increase and spread of the mutated cells compared to wild‐type cells (Goriely & Wilkie, 2012). This was further supported by the finding that mutated cells were found in clusters within the testes rather than randomly distributed (Shinde *et al*., 2013). Currently, little is known about the prevalence of germline mosaicism, how it changes with organism age and whether different germ cell lines compete with each other.

## III. MEIOTIC PHASE

During meiosis, eukaryotic chromatin is drastically reconfigured: movement and reshaping of the nucleus within the cell, movement of chromosomes within the nucleus, condensation and pairing of chromosomes, and recombination between homologous chromosomes take place in an intricate sequence with precise coordination (see Fig. 1). Variation in the cellular mechanisms that create the complex underlying apparatus at any stage may have direct implications for the fertility of the organism, the inheritance patterns of alleles and/or the fitness of the offspring. In addition, DNA within the nucleus is not randomly positioned; the organisation of chromosomes into discrete territories is well established, although how the chromosomes are positioned varies among cell types (e.g. Cremer *et al*., 2001; Tanabe *et al*., 2002). The location and relative positioning of these territories within the nucleus affects a range of processes including the rate of recombination and mutation and allows the identification of mutation and recombination hotspots. Much of the research detailed below was performed in mammals, with distinct differences observed between male and female meiosis.

### (1) Genome organisation in male meiosis

In mouse spermatocytes during prophase, telomeres are anchored at the nuclear lamina, with the chromosome forming an arc between them (Fig. 1; Berríos *et al*., 2010). This arrangement persists in mature sperm, with the centromeres aggregating in central clusters (Haaf & Ward, 1995). This chromocentric pattern is common in eutherian mammals, including pigs (*Sus scrofa domesticus*) (Bhagirath, 1990) and humans (Ioannou *et al*., 2017) among others, although not in the marsupial opossum (*Monodelphis domestica*) (Namekawa *et al*., 2007). In terms of the development of crossovers and breakpoints, the three‐dimensional (3D) organisation of the genome places constraints on which chromosomal rearrangements can occur, simply due to physical proximity.

Over the past decade, it has become clear that chromatin organisation is far more complex than just partitioning into chromosome territories. Regions of each chromosome are folded into topologically associated domains (TADs), which have cell type‐specific patterns, and allow local remodelling of chromatin accessibility and thus control of gene expression on a tissue by tissue basis. The local chromatin density separates the genome into open ‘A’ compartments and closed ‘B’ compartments. These compartments correlate with GC‐content and histone modifications, as well as DNase I hypersensitivity (Lieberman‐Aiden *et al*., 2009). Chromatin conformation capture methods and their derivatives (e.g. Hi‐C, a method to study the three‐dimensional architecture of genomes; Rao *et al*., 2014) have provided a window into the chromatin behaviour of many different cell types.

Studies of the detailed TAD structure of male gametes have been complicated by the profound condensation of the genome, which makes sperm chromatin especially refractory to the solubilising methods commonly used for chromosome conformation capture methods such as Hi‐C. It has only been comparatively recently that Hi‐C maps have been produced for sperm [in mice (Battulin *et al*., 2015; Ke *et al*., 2017)], suggesting similarities between mature sperm TAD structure and other common cell types. Recently, work in both rhesus monkey (*Macaca mulatta*) (Wang *et al*., 2019) and mouse (Alavattam *et al*., 2019; Patel *et al*., 2019) have shown that during prophase the normal TAD structure breaks down during male meiosis, and most inter‐ and intra‐chromosomal contacts are lost, yet the segregation of chromatin still closely follows its transcriptional state. Following completion of meiosis, a subset of TADs are re‐established, with similarities to their precursor state. Since crossovers appear to form preferentially in the gene‐dense ‘A’ compartment, there is a direct link with opportunities for non‐allelic recombination driven by chromatin organisation.

### (2) Genome organisation in female meiosis

In oocytes, meiotic chromatin detaches from the nuclear envelope at the diplotene stage of prophase following recombination. The chromatin compacts into a heterochromatic structure called the karyosome, or karyosphere (Gruzova, 1988). This organisation appears limited to germ cells; although it has been described (and indeed was discovered) in spermatocytes (Blackman, 1905), it is far more common in oocytes. Differences in terminology have complicated comparative descriptions [see Luciano *et al*. (2014) for a survey of germinal vesicle oocytes across mammals]. Differences in chromatin architecture correlate with altered patterns of transcriptional activity and altered metabolic properties, opening the possibility for chromatin state to be used as a marker for oocyte competence in *in‐vitro* maturation systems (Vanhoutte *et al*., 2009).

Describing the detailed TAD structure of developing oocytes has proved more complex than for spermatogenesis; the limited supply of cells has required the development of single‐cell Hi‐C methods (Flyamer *et al*., 2017). This has revealed variability in chromatin structure between individual cells, while corroborating the overall patterns of A/B compartments and TADs seen in other cell types. Strikingly, A/B compartmentalisation was absent from zygotes, indicating that TADs and A/B compartments develop *via* different pathways.

### (3) The relationship between genome organisation and germline mutation

The looping structure of TADs is primarily mediated by the CCCTC‐binding factor (CTCF) and cohesins. CTCF is a zinc‐finger protein, which binds target motifs in DNA and dimerises to bring sequentially distant loci into 3D proximity. This affects gene activity by allowing promoters and enhancers to come into association, as well as by affecting the accessibility of DNA to transcription factors (e.g. Greenwald *et al*., 2019). The points at which CTCF binds are known as loop anchor points (LAPs), and are associated with hotspots for recombination and evolutionary breakpoints (Kaiser & Semple, 2018). Cohesins maintain the cohesion of sister chromatids after DNA replication. In addition to the ubiquitous cohesins, there are (in mammals) four additional meiosis‐specific cohesins. The roles of each of these have been elucidated in mice (Biswas *et al*., 2016). For further details on the cohesin complexes in meiosis, see the recent review by Ishiguro (2019). Cohesin levels in humans decrease with increasing maternal age (Zielinska *et al*., 2015), contributing to segregation errors and increased rates of aneuploidy.

Experiments in the yeast *Saccharomyces cerevisiae* have shown different patterns. During vegetative growth, yeast chromosomes adopt the so called ‘Rabl configuration’, in which centromeres position together at one pole of the nucleus, and the telomeres lag at the other side, but this arrangement alters dramatically during meiotic prophase as chromosomes undergo recombination. Specifically, despite *S. cerevisiae* lacking CTCF, Schalbetter *et al*. (2019) reported the formation of cohesin‐dependent barriers in meiotic chromatin, which define preferred chromatin loop domains that divide the genome into distinct compartments reminiscent of, but distinct from, the mammalian TAD organisation. Diverse organisations such as these illustrate the need for further comparative genomic studies to identify the mechanistic roles of the proteins involved, as well as the importance of the 3D structure in the proper completion of meiosis.

With regard to chromosomal breakpoints and rearrangement, sperm‐ or oocyte‐specific Hi‐C maps are needed from different species to understand which loci are protected in TADs, which are consistently exposed, and which vary among species. This packaging impacts the distribution of fragile DNA sites and breakage hotspots. As evidence accumulates of ‘homologous syntenic blocks’ (Larkin *et al*., 2009) which have maintained synteny over hundreds of millions of years, it is clear that there is a selective disadvantage to disrupting some loci.

For an evolutionary understanding of the meiotic processes of chromatin organisation and homologue recognition and pairing, we need further studies of both oogenesis and spermatogenesis. How does the packaging of chromatin differ among species? Which regions of the sperm genome retain histones, and which are repackaged with protamines? Is there a relevance to which sperm chromosomes/loci are the first to enter the egg? These questions can only be answered with data from multiple lineages.

There is an incredible diversity in chromosome and genome sizes among sexual eukaryotes. For example, wheat chromosomes are over tenfold bigger than the entire genome of the model yeast *S. cerevisiae*. One contentious point is whether chromosome size and ploidy level could affect chromatin remodelling and homologue recognition and pairing, with cascade effects on chromosome segregation and gamete viability. Plant species with large chromosomes have been shown to undergo extensive chromatin remodelling at the onset of meiosis (Dawe, 1998). However, homologue recognition appears to occur in a matter of few hours, regardless of genome and chromosome sizes (Moore & Shaw, 2009). In many species, at meiosis, chromosomes cluster by their telomeric ends in a ‘bouquet’ structure which facilitates proper homologue recognition. Studies on polyploid wheat strains show that during telomere clustering, subtelomeric regions need to show critical levels of homology and sequence identity for proper chromatin remodelling and homologue pairing, and after subtelomeric pairing, homologous chromosomes ‘zip‐up’ throughout their length (Colas *et al*., 2008). However, this mode of homologue recognition found in wheat is not universal, and a diversity of recognition and pairing mechanisms seem to exist, likely affected by chromosome and genome sizes.

### (4) Selfish elements utilise microtubule modifications to cheat

Non‐Mendelian segregation – biased inheritance of specific alleles – is well known, but the mechanisms involved have only recently begun to be unravelled. Meiotic drive is used by selfish elements to enhance their transmission either by eliminating competing gametes or by exploiting the asymmetric female meiotic spindle to ensure preferential chromosome segregation to the egg. Centromeres with expansive repetitive DNA sequences can act as selfish elements and drive preferential selection (Henikoff & Malik, 2002). Differences in the abundance of these repeats between the centromeres of the homologues determine which centromere is stronger. The consequence is that the chromosome homologue with more repeats, and thus the stronger centromere, will preferentially segregate to the egg while the weaker is discarded into the polar body.

Recent intriguing mechanistic findings in mice oocytes reveal that asymmetric post‐translational microtubule modifications in the spindle during meiosis I are exploited by selfish elements (Dumont & Desai, 2012; Akera *et al*., 2017, 2019). The spindle half closest to the cortex is rich in tyrosinated alpha‐tubulin whereas the egg side of the spindle contains mainly detyrosinated alpha‐tubulin. The stronger selfish centromeres preferentially associate with detyrosinated microtubules at late metaphase I, thus ensuring their transmission to the germline. It seems that the stronger centromeres form stable attachments to detyrosinated microtubules, but only weak associations with tyrosinated microtubules. This also enables strong centromeres that have initially associated with tyrosinated spindle microtubules (closest to the cortex) to detach and flip to the egg side. This difference in attachment stability may be due to strong centromeres recruiting more kinetochore proteins than weak centromeres, which could potentially affect microtubule capture, dynamics and stability at the kinetochore. The tyrosination/detyrosination‐based spindle asymmetry is triggered by signals from the cortex that are dependent on the activity of the GTP‐binding nuclear protein Ran (RanGTP gradient) and active cell division control protein 42 (CDC42), a RhoGTPase regulator of actin filaments and microtubules, at the cortex. Inhibition of Ran (RAs‐related nuclear protein) or CDC42 eliminates spindle asymmetry and prevents biased centromere/chromosome segregation (Dumont & Desai, 2012; Akera *et al*., 2017, 2019). Selfish elements therefore seem to exploit asymmetric tubulin detyrosination of the spindle microtubules to cheat and enhance their own transmission to the next generation. It remains unclear whether this asymmetry within the spindle machinery is an evolutionarily conserved mechanism spanning the major kingdoms of living organisms, and whether spindle asymmetry shows variation across populations and species. Important insights should come from studies on hybridisation zones, where chromosomes from hybridising species compete for their transmission and maintenance in hybrid populations. Studies on mice have highlighted the importance of chromosomal rearrangement in determining centromere strength and drive direction (Chmátal *et al*., 2014), but our understanding of the natural variation of centromere strength and spindle machinery and their role in producing reproductive isolation still remains in its infancy.

### (5) Meiotic mutations

The process of meiotic recombination and chromosome segregation is inherently mutagenic, driving specific mutational signatures that are not seen in somatic cells. Meiotic mutations can be divided into four classes: (*i*) whole‐chromosome events caused by mis‐segregation during meiotic division, (*ii*) structural rearrangements caused by NAHR, (*iii*) mutations associated with gene conversion during recombination and (*iv*) point mutations. Meiotic recombination initiates by the formation of numerous programmed DNA DSBs created by the evolutionarily conserved topoisomerase‐related enzyme, meiotic recombination protein SPO11. In recent years, it has become increasingly clear that both DSB formation and repair are subject to significant layers of regulation, which collectively control the location, frequency, time and ultimately the outcome of the genetic recombination process. Below we summarise advances that have been made in elucidating these modes of regulation and how they influence accurate chromosome segregation.

#### (a) DSB location

Like patterns of genetic recombination themselves, the SPO11‐DSB precursors arise non‐randomly across the genome, being (in most organisms studied to date) focused within regions of preferred activity described as ‘hotspots’ (Lichten & Goldman, 1995). Modern methods involving deep sequencing have allowed the direct mapping of SPO11‐DSB activity across diverse eukaryotic genomes, enabling a more thorough and detailed analysis of the features that regulate the spatial patterning of recombination (e.g. Lange *et al*., 2011; Pan *et al*., 2011; Fowler *et al*., 2014; Smagulova *et al*., 2016; Choi *et al*., 2018). In single‐celled eukaryotes such as *S. cerevisiae*, hotspots are primarily located within the regions of accessible chromatin generally found at gene promoters (Pan *et al*., 2011), and are often excluded from repetitive DNA that might otherwise pose a challenge to accurate repair (Sasaki *et al*., 2013). However, for most hotspots, neither transcription rate, nor the epigenetic histone modifications (H3K4me3) that mark sites of active transcription are directly quantitatively correlated with hotspot usage (Tischfield & Keeney, 2012). Instead SPO11 and the cofactors involved in promoting DSB catalysis, are likely to be opportunistically utilising chromosomal regions that are on average more accessible to DSB formation – which, for mechanistic reasons, are frequently the regulatory elements of genes. As described above, higher order chromatin structure also shapes hotspot usage: during prophase chromosomes assemble into linear arrays of packed loops, embedded at their base within the proteinaceous axial core of the chromosome (Fig. 1), and it is within this context that recombination initiates. As a result, even sites of open chromatin become inefficient sites for SPO11‐DSB formation should they arise in locations that frequently are embedded in the axis at the base of such loops (Ito *et al*., 2014). In some, but not all, multicellular eukaryotes much of this basal regulation is over‐written *via* the activity of the multifunctional protein, PR domain zinc finger protein 9 (PRDM9) – a ‘Swiss army knife’ of the recombination process, which, *via* its DNA binding domain and histone trimethylation (H3K4me3) activity, influences the location and efficiency of DSB formation and repair (Borde *et al*., 2009; Neale, 2010; Baker *et al*., 2017; Grey, Baudat & de Massy, 2018). Moreover, because the DNA binding motif of PRDM9 evolves rapidly, individuals bearing different PRDM9 alleles can display very different patterns of recombination (Neale, 2010; Grey *et al*., 2018). Following DSB formation, pairing (mis‐pairing in the case of NAHR) and recombination take place.

#### (b) Timing and frequency

Whilst every genome contains many thousands of potential sites where DSBs can initiate recombination, only a subset of these are used in each meiotic cell. In general, recombination frequency increases with genome size, but does not necessarily scale linearly. In the small genomes of single‐celled eukaryotes, DSB frequency ranges from ~60 (*Schizosaccharomyces pombe*) to ~200 (in *S. cerevisiae*) per genome. By contrast, DSB frequency in *Arabidopsis thaliana* is ~300 DSBs, and in the larger genomes of mice and humans is ~500 DSBs per genome, despite a genome ~250 times larger than yeast. What controls DSB and recombination frequency is of considerable interest. Recent findings support a network of antagonistic regulatory pathways (Cooper *et al*., 2014; Cooper, Garcia & Neale, 2016). Cyclin‐kinase activity appears first to licence the process of DSB formation following local DNA replication (Henderson *et al*., 2006; Sasanuma *et al*., 2008; Borde *et al*., 2009; Murakami & Keeney, 2014). This pro‐DSB stage is maintained by transient checkpoint‐dependent arrest in meiotic prophase triggered by the ongoing recombination process, thereby permitting time for DSBs to be formed across all chromosomes (Gray *et al*., 2013). Exit from this DSB‐competent stage appears to be achieved in several ways: firstly by DSB repair dampening the checkpoint signal, enabling exit from meiotic prophase (Carballo *et al*., 2013; Gray *et al*., 2013), and secondly *via* the process of homologue engagement (Thacker *et al*., 2014), a somewhat unexplored mechanism in which recombination‐dependent interactions between homologous chromosomes locally suppress SPO11 potential. At least in *S. cerevisiae*, higher order chromosome architecture also influences recombination potential, with some chromosomes (mostly shorter chromosomes) experiencing earlier and more substantial DSB activity than larger chromosomes (Pan *et al*., 2011; Murakami *et al*., 2018). Subtelomeric regions also experience elevated DSB frequency (Subramanian *et al*., 2019), consistent with reduced levels of compaction compared to interstitial regions within the chromosome arm (Schalbetter *et al*., 2017). Recombination frequency is further influenced by DSB‐dependent negative regulation; a process that appears conserved across all eukaryotes examined so far (Joyce *et al*., 2011; Lange *et al*., 2011; Carballo *et al*., 2013; Garcia *et al*., 2015; Mohibullah & Keeney, 2017). Abrogation of these surveillance mechanisms causes both increases in SPO11‐DSB formation, and loss of spatial constraints, thereby enabling recombination events to initiate in close proximity to one another (Garcia *et al*., 2015), potentially complicating the fidelity of downstream repair processes.

#### (c) Repair and segregation

Once formed, all SPO11‐DSBs must repair prior to the onset of meiotic chromosome segregation. In most, but not all, organisms, preferential repair using the homologous chromosome is critical to promote homologue pairing and the generation of interhomologue crossover events. Such regulation depends on the structure of the chromosome axis, including factors such as the meiotic cohesin subunit, Rec8 (Kim *et al*., 2010), and HORMA–domain proteins (i.e. as found in HOP1P, REV7P and MAD2) and their partner proteins (RED1, MEK1, HORMAD1 etc; Schwacha & Kleckner, 1997; Carballo *et al*., 2008; Callender & Hollingsworth, 2010; Daniel *et al*., 2011), as well the active recombination machinery (RAD51 and DMC1; Hong *et al*., 2013; Lao *et al*., 2013), and sensory kinases such as ATM (ataxia telangiectasia mutated protein) and ATR (ataxia Rad3‐related) (Grushcow *et al*., 1999; Carballo *et al*., 2008). In *S. cerevisiae* the efficiency of homologue repair increases over time (Joshi *et al*., 2015). Early, low‐abundance DSBs show preferential repair with the sister chromatid rather than with the homologous arm. With time, the gradual increase in DSB density results in an increase in homologue pairing and repair *via* recruitment of an alternative serine/threonine‐protein kinase MEC1‐dependent pathway. These findings suggest that DSB repair with the sister chromatid is the default pathway and its existence could provide some protection from DSB damage (Joshi *et al*., 2015).

The repair of DSBs by inter‐homologue recombination does not always generate reciprocal exchanges of chromosome arms (crossovers). A large fraction of DSBs are repaired by producing recombinant molecules *via* gene conversion without crossover (non‐crossovers). Non‐crossovers are believed to be 10 times more frequent than crossovers from studies in mice and humans (Baudat, Imai & de Massy, 2013). The fraction of recombination intermediates resulting in crossovers varies among organisms, from as high as 50% in *S. cerevisiae* to ~10% in higher eukaryotes such as mammals and plants (Novak, Ross‐Macdonald & Roeder, 2001; Baudat & de Massy, 2007). Crossovers themselves are also classified into those which do and do not display interference – an evolutionarily conserved process that prevents two or more crossovers from forming near one another along chromosomes (de los Santos *et al*., 2003; Hollingsworth & Brill, 2004; Mezard *et al*., 2007). Current models suggest an interplay between stress caused by chromosome axial compaction (Kleckner *et al*., 2004; Zhang *et al*., 2014), and gradual maturation of crossover‐designated precursors (Holloway *et al*., 2014) as fundamental to the interference process.

In mammals, especially humans, crossover designation and distribution are particularly important to meiotic success. Recent work suggests that the higher rates of chromosome mis‐segregation in female meiosis can be explained by inefficient crossover maturation during meiosis I (Wang *et al*., 2017). Chromosome mis‐segregation seems particularly common in human female meiosis, and is associated with maternal age, the higher the age of the mother, the higher the risk of chromosome mis‐segregation and thus aneuploidy. Aneuploidy is much more common on short and/or acrocentric chromosomes since inefficient cross‐over maturation will have more dramatic effects in such cases (Wang *et al*., 2017). A typical example is the mis‐segregation of chromosome 21 leading to Down syndrome. The evolutionary significance of this inefficient chromosome segregation associated typically with humans, and specifically with female meiosis remains to be understood, but the general view is that this could be an evolutionarily selected trait. Increased aneuploidy would lead to zygote inviability, increased implantation failure and miscarriage, which in turn would increase the time between pregnancies and decrease the likelihood of pregnancy in older women. Both life‐history traits should guarantee improved parental investment, which has been critical to the evolutionary success of the human species (Westendorp & Kirkwood, 1998).

### (6) Non‐allelic homologous recombination

NAHR is a prominent source of structural genomic variation and contributor to *de novo* inherited disease. NAHR requires two successive processes: initiation and resolution of recombination within pre‐existing homologous sequences (e.g. genomic repeats), and may lead to inversions, deletions, insertions or duplications of the intervening sequence between the recombining loci. Similar to homologous recombination NAHR also uses DSB sites and PRDM9 motifs. The effects of PRDM9‐mediated control of recombination hotspots have been best characterised in mammals, where the association between recombination and PRDM9 activity explains the presence of the PRDM9 binding motif in pathogenic NAHR‐related rearrangements arising during meiosis (e.g. Hillmer *et al*., 2016). Since PRDM9 is not expressed in somatic cells, this signature is not seen in rearrangements arising from somatic NAHR (Vogt *et al*., 2012). Following DSB formation, pairing (mis‐pairing in the case of NAHR) and recombination take place, however, the factors regulating mis‐pairing between non‐allelic repeat units remain to be clarified. We therefore expect further genetic factors to be identified in the future (Liu *et al*., 2011; MacArthur *et al*., 2014).

Gene conversion arises due to mismatch repair or base excision repair of heteroduplex tracts generated during strand invasion and recombination. There are two significant biases to consider here. The first gene conversion bias acts in favour of gene conversion of the invading strand, i.e. the strand in which the meiotic DSB initially formed (Nicolas *et al*., 1989; Webb, Berg & Jeffreys, 2008). This means that alleles that are prone to DSB formation, such as PRDM9 binding sites in humans and mice, will preferentially be converted into insensitive alleles with a lower DSB formation rate, a process known as ‘hotspot erosion’. Several lines of evidence suggest that in mammals and other taxa with PRDM9‐directed recombination, hotspot erosion is a major driver of PRDM9 gene sequence evolution, hybrid incompatibility between species, and speciation (Oliver *et al*., 2009; Lesecque *et al*., 2014; Smagulova *et al*., 2016). The second gene conversion bias is a generalised bias in favour of GC alleles over AT alleles, arising *via* long‐patch mismatch repair (MMR) at crossover‐associated heteroduplexes (Duret & Galtier, 2009; Lesecque, Mouchiroud & Duret, 2013). This drives GC accumulation in highly recombinogenic areas of the genome such as avian microchromosomes (Webster, Axelsson & Ellegren, 2006), sex chromosome pseudoautosomal regions (Raudsepp & Chowdhary, 2016) and recombination hotspots in multiple taxa (Sundararajan *et al*., 2016). Over evolutionary time, this GC bias ultimately leads to the stratified isochore structure of eukaryotic genomes (Duret *et al*., 2002). A recent suggestion is that GC bias during mismatch repair is driven by a selective pressure to reduce mitotic mutations occurring as a result of spontaneous cytosine deamination (see Section II.1), and that GC‐biased gene conversion is simply a side effect of this process (Lesecque *et al*., 2013).

An intriguing question for future research is the interplay between these two forms of biased gene conversion. In taxa without PRDM9‐directed hotspot formation such as birds and yeast, recombination preferentially occurs at gene promoters. Conversely, in taxa with PRDM9‐directed hotspot formation, recombination preferentially occurs in intergenic regions marked by PRDM9 motifs. Is the recurrent gain/loss of PRDM9 involvement in recombination hotspot localisation a strategy to reduce/enhance the amount of GC‐biased gene conversion in promoter regions? Recently, Halldorsson *et al*. (2019) obtained high‐resolution recombination maps from almost 3000 Islandic human trios (parents and child genotypes) and found a 50‐fold increase in *de novo* mutations within 1 Kb of crossovers. This study clearly shows that meiotic mutations are not produced exclusively by gene conversion, and reveals a much greater role of crossover in mutagenesis than previously appreciated.

### (7) Variation in meiotic recombination rates

Recombination is essential for promoting accurate chromosome segregation during meiosis and generating genetic diversity. By contrast, recombination can also break apart favourable allele combinations. A trade‐off between advantageous and disadvantageous effects of recombination must therefore exist that ultimately will affect the frequencies of crossing overs during meiosis in populations at equilibrium.

Given the great variation in most traits across eukaryotic taxa, it is perhaps not surprising to find large differences in recombination rates among species (Stapley *et al*., 2017). There is consensus about the idea that natural and sexual selection may have a profound effect on the rate of recombination, both directly and indirectly (reviewed by Stapley *et al*., 2017), but the lack of coordination between theoretical and empirical studies has made rigorous testing of theoretical predictions challenging (Dapper & Payseur, 2017).

An important component of variability of recombination rates within species is the uneven distribution of crossovers across the genome. As introduced above, since the early pioneering studies on yeast (Petes, Malone & Symington, 1991), it has become clear that in a variety of organisms, crossover locations are not evenly distributed along the chromosomes, and regions of high and low recombination exist, named ‘hot’ and ‘cold’ spots, respectively. Several human hotspots have been mapped to kilobase resolution using sperm typing (Holloway, Lawson & Jeffreys, 2006).

Another important component of variation within species is the differences in recombination rates and crossover distributions between the sexes (heterochiasmy). Sexual dimorphism of meiotic recombination can have several degrees of intensity, to the extent that one sex shows free meiotic recombination across almost the entire length of the chromosomes, whereas the other completely lacks recombination (achiasmy). According to the ‘Haldane–Huxley’ rule, when achiasmy occurs, it is the heterogametic sex that is achiasmate. Notable examples of achiasmatic species are *Drosophila* and butterflies. Several hypotheses have been put forward for the existence of sexual dimorphism of recombination, generally varying depending on whether it is a case of achiasmy or heterochiasmy. Some rigorous theoretical approaches have been suggested (e.g. Burt, Bell & Harvey, 1991; Lenormand, 2003), one of which suggests a role of post‐meiotic haploid selection in the evolution of heterochiasmy (Lenormand & Dutheil, 2005).

However, a general consensus on the evolutionary drivers is still lacking and empirical evidence to test theoretical predictions is scarce (Lenormand & Dutheil, 2005). Gaining information on recombination rates from cytogenetics, pedigrees and genetic maps is the first step in elucidating the molecular basis and the evolutionary forces underlying variation in recombination rates, but these procedures are time consuming and present technical and biological difficulties. The introduction of genome‐wide sequencing techniques has made it feasible to obtain thousands of markers for a fine‐scale resolution of recombination landscapes across the genome. These include gamete‐typing approaches which are potentially very promising in estimating sex differences in recombination rates (Ottolini *et al*., 2015, 2016; Dréau *et al*., 2019). Similarly, approaches allowing robust linkage mapping even for low‐coverage whole‐genome data (Rastas, 2017) will help to address some of the gaps mentioned above.

Alternative approaches that use genomic population data have also been developed, based on measures of linkage disequilibrium (Jeffreys, Murray & Neumann, 1998; Jeffreys, Ritchie & Neumann, 2000; Crawford *et al*., 2004; Brunschwig *et al*., 2012). Caveats in the use of linkage disequilibrium have been tackled by Hedrick (1987). The use of linkage disequilibrium from genomic data has resulted in the production of genomic landscapes of recombination rates and hotspots. These studies have highlighted the extreme variation of hotspot distribution among closely related species, but also polymorphisms within species, thus advancing our understanding of the extent of recombination rate variation in natural populations (Myers, 2005; Brunschwig *et al*., 2012).

Once recombination landscapes have been determined under different regimes of natural and/or sexual selection, a better understanding of the molecular determinants and evolutionary drivers that mediate crossing‐over differences within and among species can be achieved. Some progress towards elucidating the molecular mechanisms at the basis of recombination variation has started to emerge (Zelkowski *et al*., 2019). A recent study on two species of *Drosophila* revealed a meiosis gene acting as a modifier of species differences in crossing over (Brand *et al*., 2018). The authors hypothesised that the rapid evolutionary rates in this particular gene may be directly linked with recurrent bouts of adaptive evolution in a coevolutionary arms race with selfish genetic elements. Kent, Uzunovic & Wright (2017) also review the evidence for the typically negative association between TEs and meiotic recombination rates. While traditionally this negative association was thought to be a result of increased accumulation of TEs in regions with low recombination rates, regulation of TE activity could directly affect recombination rate (Kent *et al*., 2017). Finally, the previously mentioned meiotic gene *PRDM9* is regarded to have an important role in specifying recombination hotspots in many metazoans, and its rapid evolution could be correlated with the extreme turnover of hotspots (Ségurel, Leffler & Przeworski, 2011; Úbeda, Russell & Jansen, 2019).

## IV. POST‐MEIOTIC PHASE

The window of time between the end of meiosis and the moment of nuclear fusion during fertilisation is potentially short, particularly in diplontic organisms, but processes occurring at this stage are key in determining the genetic variation in the next generation(s) (Immler, 2019; Fig. 1).

### (1) Post‐meiotic mutations

Post‐meiotic cells, in particular highly differentiated male gametes in taxa with anisogamy, have further unique mutational pressures to consider. Their haploid nature means that there is no template available for homologous repair, and all DSBs must therefore be repaired by some other mechanism – primarily non‐homologous end joining (NHEJ) in spermatids (Leduc *et al*., 2008; Ahmed, Scherthan & de Rooij, 2015). Importantly, the high degree of chromatin compaction in mature sperm involves a genome‐wide wave of DSB formation, most likely as a result of topoisomerase activity to relieve DNA helix torsion. These post‐meiotic DSBs have recently been shown to occur in discrete hotspots in mouse, yeast and *Tetrahymena*, and may be enzymatically induced (Akematsu *et al*., 2017; Cavé *et al*., 2018; Grégoire *et al*., 2018).

The mutational consequences of these post‐meiotic DSB hotspots are as yet unknown, and thus raise a number of fundamental questions. For example, are hotspots conserved across species, do they show signatures of elevated NHEJ‐mediated mutagenesis, and do they act as the nuclei for wider structural rearrangements? There is evidence that NHEJ‐mediated repair of post‐meiotic DSBs is one mechanism leading to paternal‐specific germline expansion of Huntington disease‐associated trinucleotide repeats, in addition to the better‐known mechanism of slippage during replication (Garcia‐Diaz & Kunkel, 2006; Simard *et al*., 2014). Does this finding also apply to other types of NHEJ‐mediated genomic rearrangement such as translocations and inversions with breakpoints in single‐copy regions? Might other forms of repair such as break‐induced replication (BIR) also be important in this context, and can post‐meiotic DSBs trigger more complex genomic rearrangements and copy‐number changes? How do these DSB hotspots relate to chromatin organisation and histone modification in gametes?

A final mutational pressure specific to male gametes is the consequences of cytoplasm reduction. This leaves these gametes lacking in antioxidants and repair enzymes, leaving them vulnerable to oxidative damage to DNA. As with post‐meiotic DSBs, oxidative damage has also been shown to be localised into hotspots (Kocer *et al*., 2015), and it is not yet known whether these are also mutational hotspots (see Long *et al*., 2016). Since oxidative damage is in general not repaired in the mature gamete, but instead is repaired in the zygote post‐fertilisation, much will depend on the specific mechanisms available for this repair and the degree of damage that can be tolerated (Garcia‐Rodriguez *et al*., 2019).

### (2) Post‐meiotic selection

The haploid state of post‐meiotic cells potentially opens a particularly sensitive window for selection to act upon. Several post‐meiotic drive systems are known to take advantage of haploid gene expression, such as the *t*‐haplotype (Lyon, 2003) in the house mouse, and *Segregation Distorter* (*SD*) gene complex (Larracuente & Presgraves, 2012) and Winters (Tao *et al*., 2007) in *Drosophila* fruitflies. Apart from these examples of extreme effects, any genes expressed in a haploid state may be under selection without the possible masking effects of any sister alleles. In plants, haploid gene expression and selection has long been known (Mulcahy & Mulcahy, 1975) as pollen tubes compete for ovules (Erbar, 2003), and recent studies provided clear evidence for positive and purifying selection in genes expressed in the haploid stages (Arunkumar *et al*., 2013). By contrast, in animals, selection occurring at the haploid stage has long been dismissed. In females, meiotic divisions generally occur only a short time before fertilisation – indeed fertilisation is often the trigger for the second meiotic division. Moreover, there is no interphase between female meiotic divisions, and the chromatin remains tightly condensed and transcriptionally inactive. It is thus currently believed that there is little or no opportunity for haploid selection between female gametes, and the study of female transmission ratio distortion has focused on genes that ‘cheat’ during meiotic division and compete to be included in the oocyte rather than the polar body (see Section III.4). By contrast, haploid male germ cells have an extended developmental window following the meiotic divisions during which morphologically unspecialised round spermatids are remodelled into mature sperm, and subsequently an extended free‐living stage. Haploid selection may potentially operate during both of these windows.

However, the scope for haploid selection in developing spermatids is markedly restricted by the fact that male germ cells remain functionally linked through cytoplasmic bridges throughout development, and share transcripts, gene products and even whole organelles between the haploid post‐meiotic cells (Dym & Fawcett, 1971; Caldwell & Handel, 1991). This is a fundamental feature of male meiosis but may be particularly important in species with genetic sex determination, where transcripts from different sex chromosomes need sharing across spermatids to avoid strong sex‐biased expression at the post‐meiotic stages. However, allele‐specific expression and imperfect sharing among haploid spermatids has been demonstrated for the SPAM1 (sperm adhesion molecule 1) and SMOK (sperm motility kinase) genes in house mice (Zheng, Deng & Martin‐DeLeon, 2001; Veron *et al*., 2009). Both of these were identified based on the transmission ratio distortion exhibited in males heterozygous for chromosomal rearrangements bearing mutant gene copies; the lack of sharing generates two classes of gametes that are functionally different. In the absence of linked chromosomal rearrangements, transmission ratio distortion for individual alleles will likely go unnoticed except as accelerated selection on male germline‐expressed genes.

In mature sperm, the bridges between sister cells have broken, and thus there is even more scope for competition among cells. However, the dense packing of the genome required for hydrodynamic swimming efficiency is believed to prevent active transcription during the free‐living portion of a sperm's life cycle. This condensation is achieved during the final period of spermatid development, spermiogenesis, during which (in most species) histones are replaced with smaller protamines. The replacement enables a very highly compacted, toroidal, quasi‐crystalline chromatin arrangement, which is further stabilised by cross‐linking of protamine molecules. However, a certain percentage of histones are retained in sperm chromatin, ranging from 1 ∼ 8% in mouse and 10 ∼ 15% in humans up to 100% in the zebrafish (*Danio rerio*) (Hammoud *et al*., 2009; Brykczynska *et al*., 2010; Carrell, 2011; Jung *et al*., 2017). In humans, H4 histones are mostly found in distal intergenic regions of the genome whereas modified histones are enriched at specific genomic elements including H3K4m3 at CpG‐rich promoters and H3K9me3 in satellite repeats (Yamaguchi *et al*., 2018). These findings imply the existence of a machinery to keep modified histones in place, possibly because of the important role that genes near modified histones seem to play during early embryonic development (Bernstein *et al*., 2006). Whether there is any scope for these regions to remain transcriptionally active in mature sperm, particularly in species such as zebrafish that lack protamines, remains unknown.

Despite the obstacles to haploid selection imposed by transcript sharing in spermatids and genome compaction in mature sperm, the extensive literature on transmission ratio distortion across multiple taxa from fruitflies to mammals implies that haploid selection in animals may be more common than usually appreciated (Joseph & Kirkpatrick, 2004; Immler & Otto, 2018; Immler, 2019). In fact, a recent study showed that the sharing of genes across haploid spermatids is incomplete for a large number of genes in the house mouse and a primate (K. Bhutani, K. Stansifer, L. Ticau, L. Bojic, C. Villani, J. Slisz, C. Cremers, C. Roy, J. Donovan, B. Fiske & R. Friedman, in preparation). Findings in fish and mammals further suggest that the haploid genotype may contribute not only to variation in sperm phenotypes, but also considerably to the phenotypic variation encountered in the resulting offspring (Alavioon *et al*., 2017; Borowsky *et al*., 2018; Rathje *et al*., 2019). One study in the zebrafish not only presented a clear link between selection for a specific sperm phenotype and offspring fitness but linked the selected sperm phenotypes to their haploid genotypes (Alavioon *et al*., 2017). Similarly, a study using two species of *Astyanax* cavefish provided further evidence for a direct link between the haploid sperm genotypes and distinct sperm phenotypes (Borowsky *et al*., 2018). There is thus a clear need for further experimental work to tease apart the mechanisms of transcript sharing and whether the chromatin structure of mature sperm is ever permissive for transcription, and how transcriptional differences among individual post‐meiotic cells during development are parlayed into selectable functional differences among gametes.

## V. FUTURE DIRECTIONS

### (1) Genome reorganisation

Key challenges for the future include understanding the interactions between the cytoskeleton and the nucleus in regulating genome structure during meiosis. There is still a poor understanding of how meiosis differs from mitosis and of the regulators involved in each process. Specifically, we need a greater understanding of epigenetic regulation, which appears to mark genes poised for transition from mitotic spermatogonial proliferation to meiotic division (Gleason *et al*., 2018). The study of epigenetics requires the development of improved methodologies for visualising meiotic processes. Recent advances have shown promise, such as improved methods for visualising meiotic cells (Hwang, Hopkins & Jordan, 2018), new tools to study chromosome dynamics (such as the live imaging of Enguita‐Marruedo *et al*., 2018), and new tools to study the mechanisms underpinning meiotic chromosome conformation (Schalbetter *et al*., 2019).

### (2) Mutation

The study of mutational spectra in both somatic and germ cells is a rapidly expanding field. A key task for future research will be to integrate our growing knowledge of the mutation spectrum with the evolutionary study of how those mutations subsequently spread within the population. One current puzzle, for example, is the absence of the signature of APOBEC‐driven mutation in humans at the population level in studies based on trio sequencing (UK10K Consortium *et al*., 2016; Harris & Pritchard, 2017; Narasimhan *et al*., 2017). Does this indicate that population‐specific mutagenic pressures need to be accounted for in human phylogenetic studies? Or is it simply a matter of statistics? If APOBEC‐damaged gametes are rare then they might not be observed in a small‐scale survey of trios. A related question is then: what is the distribution of *de novo* mutations among individuals? Do all offspring carry a similar number of mutations, or conversely, are there occasionally viable offspring with an above‐average mutation load? Such cases of increased mutation load could result from an oxidised sperm cell, or an egg that suffered APOBEC activation at the wrong moment. Work from cancer cells suggests that mutations can cluster both in time and space. In other words, concerted formation of multiple mutations may occur simultaneously across a linked genomic region (clustering in space), or a transient induction of hypermutability without loss of fitness (clustering in time) (Roberts & Gordenin, 2014). Understanding how these mutational processes play out in the germline is key to understanding how genetic novelty is introduced to the population as a whole.

### (3) Recombination

A step forward in elucidating the significance of recombination and the causes of its variation will be the study of recombination patterns and variation within species. This will allow us to answer whether variation in recombination rates is heritable. It is an important question to answer because it will help us to predict whether recombination rates can evolve over time. Ultimately, this information could shed light on the causes of divergence of recombination rates among populations and closely related species, but more generally, it would contribute to deciphering the causes of variation and the evolutionary significance of sex and recombination.

### (4) Selection

Our understanding of selection acting on germ cells and mature gametes is still limited. Tracking *de novo* mutations among germ cell lineages and assessing their occurrence over time is now possible and will provide a higher resolution image of the dynamics occurring among germ cell lineages. Furthermore, single cell sequencing has the potential rapidly to improve our understanding of the genetic variation among germ cells and gametes and of the variation in gamete pools. Comparing this to the genetic variation in offspring will provide further information about possible selection events before, during and after meiosis.

## VI. CONCLUSIONS

1. Germline *de novo* mutations are an important source of genetic variation; the three main underlying mechanisms are DNA synthesis errors, C‐to‐T transitions, and transcription‐associated changes.
2. Another major germline‐specific source of mutations is the mobilisation of TEs and ERVs. Recent technological advances in sequencing have provided novel insights from cell lines, including cancer cell lines, and somatic development, complemented by direct studies of germline‐specific mobilisation in some model organisms.
3. Germline selection affects the spread and possible inheritance of germline‐specific *de novo* mutations, but relatively little is currently known about the exact mechanisms involved.
4. Chromatin reconfiguration occurring during meiosis is a major source of novel genotypes and includes reshaping of the nucleus, chromosome movement in the nucleus, chromosome condensation and pairing and recombination. Chromatin organisation is more complex than previously assumed, and understanding the fine detail will be key. Chromatin conformation capture methods have provided novel insights.
5. TADs have been found to play a key role in contributing to *de novo* mutations generated during meiosis. In particular, their association with CTCFs and cohesins are key to identifying breakpoint hotspots. Hi‐C maps from different species are needed to investigate this further.
6. During meiosis, microtubules play a key role in separating homologous chromosomes, and the mechanisms can be hijacked by TEs and ERVs. Oogenesis is particularly vulnerable to such asymmetric post‐translational microtubule modifications.
7. Meiotic mutations are caused by four main mechanisms: whole‐chromosome events caused by mis‐segregation during meiotic division, structural rearrangements caused by NAHR, mutations associated with gene conversion during recombination, and point mutations.
8. DSB hotspots have been identified around the SPO11‐DSB precursors as part of the genetic recombination mechanism with the help of novel deep‐sequencing methods and thorough mapping of SPO11‐DSB activity. In addition, repair of SPO11‐DSBs often do not result in reciprocal exchanges of chromosome arms but may lead to gene conversion and may result in mis‐segregation.
9. NAHR is a prominent source of structural genomic variation and contributor to *de novo* inherited disease. Similar to homologous recombination, NAHR also uses DSB sites and PRDM9 motifs. Gene conversion arises due to mismatch repair or base excision repair of heteroduplex tracts generated during strand invasion and recombination.
10. An important component of variability of recombination rates within species is the uneven distribution of crossovers across the genome. Crossover locations are not evenly distributed along the chromosomes, and regions of high and low recombination exist, named ‘hot’ and ‘cold’ spots, respectively. Another important component of variation within species is differences in recombination rates and crossover distributions between the sexes (heterochiasmy).
11. Post‐meiotic mutations are particularly important as no template is available for homologous repair. These are primarily caused by the high degree of chromatic compaction in mature sperm inducing DSBs. Alternative repair mechanisms (e.g. NHEJ) are in place.
12. Genes expressed at the post‐meiotic haploid stages may be exposed to efficient purifying and positive selection. Although transcripts are shared among haploid spermatids, sharing is not always perfect and evidence for selection at this haploid stage is accumulating rapidly.
